# Multiscale and multidisciplinary analysis of aging processes in bone

**DOI:** 10.1038/s41514-024-00156-2

**Published:** 2024-06-15

**Authors:** Linda Ravazzano, Graziana Colaianni, Anna Tarakanova, Yu-Bai Xiao, Maria Grano, Flavia Libonati

**Affiliations:** 1 Center for Nano Science and Technology@PoliMi, Istituto Italiano di Tecnologia, Via Rubattino 81, Milano, 20134 Italy; 2 Department of Precision and Regenerative Medicine and Ionian Area (DiMePRe-J), University of Bari Aldo Moro, Piazza Giulio Cesare 11, Bari, 70124 Italy; 3 School of Mechanical, Aerospace, and Manufacturing Engineering, University of Connecticut, 191 Auditorium Road, Unit 3139, Storrs, 06269 CT USA; 4 Department of Biomedical Engineering, University of Connecticut, 260 Glenbrook Road, Unit 3247, CT 06269 Storrs, USA; 5 Department of Mechanical, Energy, Management and Transport Engineering - DIME, University of Genova, Via all’Opera Pia 15, Genova, 16145 Italy

**Keywords:** Chemistry, Molecular biology, Medical research

## Abstract

The world population is increasingly aging, deeply affecting our society by challenging our healthcare systems and presenting an economic burden, thus turning the spotlight on aging-related diseases: exempli gratia, osteoporosis, a silent disease until you suddenly break a bone. The increase in bone fracture risk with age is generally associated with a loss of bone mass and an alteration in the skeletal architecture. However, such changes cannot fully explain increased fragility with age. To successfully tackle age-related bone diseases, it is paramount to comprehensively understand the fundamental mechanisms responsible for tissue degeneration. Aging mechanisms persist at multiple length scales within the complex hierarchical bone structure, raising the need for a multiscale and multidisciplinary approach to resolve them. This paper aims to provide an overarching analysis of aging processes in bone and to review the most prominent outcomes of bone aging. A systematic description of different length scales, highlighting the corresponding techniques adopted at each scale and motivating the need for combining diverse techniques, is provided to get a comprehensive description of the multi-physics phenomena involved.

## Introduction

**Fig. 1:**
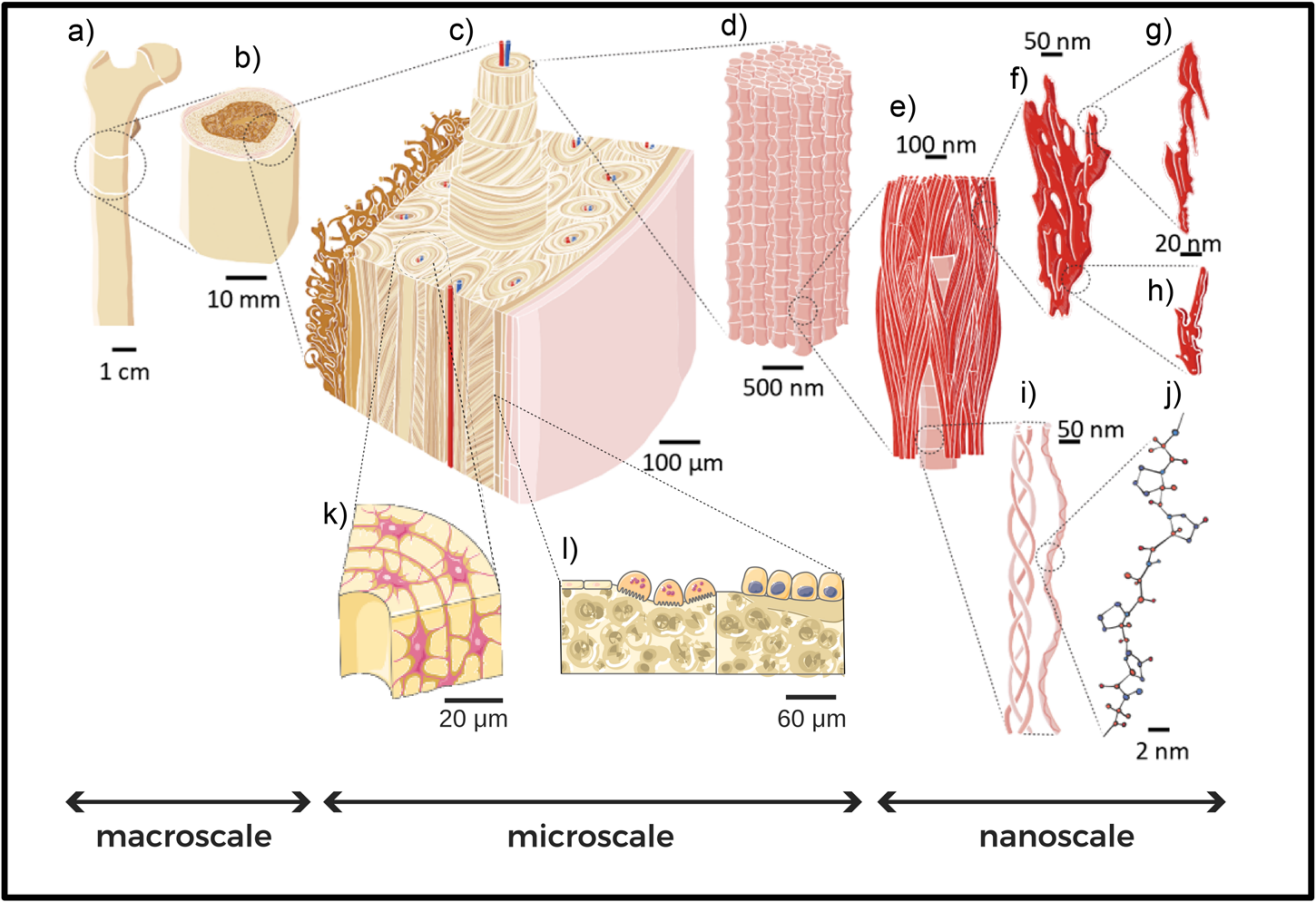
Multiscale structure of bone. **a** Human femur as an example of bone; **b** femur cross section showing the cortical tissue (outer layer) and the trabecular tissue that accomodate bone marrow (inner part); **c** magnification of the previous panel, with a focus on the Haversian structure: the main building block is the osteon, a hollow composite cylinder, made of several concentric fiber-reinforced layers, the lamellae, each one showing a preferential orientation of the mineralized collagen fibers; **d** collagen fiber, represented as a collagen fibril bundle (the surrounding matrix is removed for clarity); **e** mineralized collagen fibril, base constituent of the collagen fiber, which includes both intra- and extra-fibrillar hydroxyapatite (HA) mineral crystals; **f** mineral aggregate, including HA in both the forms of **g**) platelet and **h**) acicular crystals; **i** molecular component of the collagen fibers, aka tropocollagen, consisting of three polypeptide chains coiled around each other; **j** magnification of a polypeptide chain; **k** focus on the osteon structure, to reveal the osteocytes lying inside lacunae in the extracellular matrix of bones. Lacunocanalicular networks formed by dendrites propagating from osteocytes is clearly visible; **l** focus on bone cells responsible for the continous remodeling process. In particular, osteoclasts are responsible for bone resorption (on the left) and osteoblasts are involved in bone formation (right). The whole figure is adapted from Gabriele Grezzana et al. “Probing the Role of Bone Lamellar Patterns through Collagen Microarchitecture Mapping, Numerical Modeling, and 3D-Printing.” Advanced Engineering Materials 22.10 (2020): 2000387. Copyright 2020, with permission from Elsevier^133^. Subfigure (**k**) and (**l**) are built with Servier Medical Art.

**Fig. 2:**
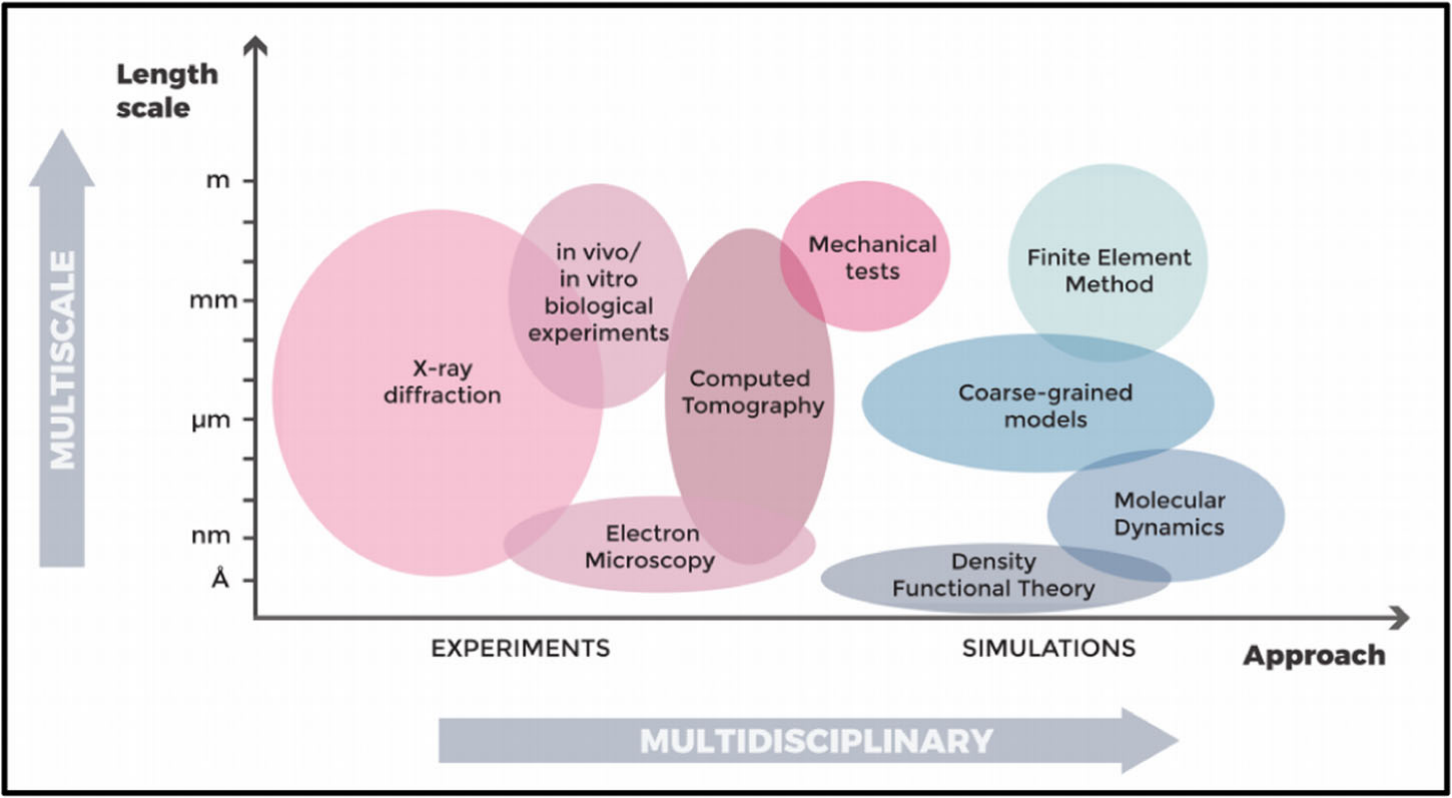
Multiscale and multidisciplinary approaches used to study bone aging. Overview of experimental and computational techniques suitable for studying various aspects of the aging process in bone tissue. Each “bubble” represents a specific approach and the “bubble” height spans the length scale of its applicability.

According to the 2019 United Nations data, the elder population is increasing in almost all countries. Currently, 9% of the world population is over 65 and that percentage will increase up to 16% by 2050^1^. This evolution will profoundly impact our society, challenging our healthcare systems, and bringing the necessity of redesigning our healthcare services to accommodate the needs of the increasing number of geriatric patients^2^ and guaranteeing them a better life quality. Among the several degenerative and chronic pathologies and conditions associated with aging, a prominent role is played by bone and skeletal diseases. Osteoporosis is a widespread pathological condition that affects more than 200 million people worldwide and is associated with aging^3^. Osteoporosis and, more generally, the age-related degradation of bone tissue, causes a significant increase in fracture risk for the elderly. Indeed, age-specific rates of fracture incidence are higher in the oldest age groups, affecting more than 15% of those aged 95 and above^4^. From this perspective, more widespread injury-prevention efforts and access to screening and treatment of osteoporosis for older individuals should help one to reduce the overall burden and improve patient and family lives^4^. In order to look at bone fragility in a wider perspective^5^ and to successfully tackle age-related bone diseases, it is crucial to deeply understand the fundamental mechanisms responsible for bone tissue degradation. Indeed, bone is a complex multiscale composite material, in which organic and inorganic components arrange themselves following specific patterns and structures at different length scales. The inorganic mineral hydroxyapatite crystals (see Fig. 1f, g and h) and the organic collagen molecules (see Fig. 1i, j) constitute the main building blocks of bone. These fundamentals “bricks” of bone arrange themselves to form mineralized collagen fibrils (see Fig. 1d). Fibrils form more complex structures going up in scale (in a certain way reminiscent of a fractal geometry), such as fibers and bundles of fibers. The latter are arranged, by following diverse orientations, depending on the anatomical area, into arrays and lamellae (at submiscroscale). During remodeling, lamellae circumferentially arrange to form cylindrical features, called osteons (see Fig. 1c), which are interspersed into interstitial lamellae, forming bone extracellular matrix. Inside the complex structure of the bone matrix, a variety of bone cells live and play a fundamental and active role in bone growth and remodeling (see Fig. 1k, l). All those elements play a role in determining the features and properties of bone tissue at the meso- and macro-scale (see Fig. 1b and a, respectively). It is clear how a systematic study of the aging processes in bone should embrace this complexity and carefully take care of the changes that aging causes at each level of bone’s hierarchical structure, pursuing a multiscale approach. Recent works have covered different aspects of bone aging, such as changes in the nanoscale bone components^6^, the impact on bone cellular processes with consequences on bone tissue^7^, and the variation of bone mechanical properties^8^. In some cases, a multiscale approach has been adopted^7–10^, but keeping the focus on a specific topic related to bone aging^7,8^, or using a specific technique^10^. Indeed, the literature scenario on bone aging is quite ‘fragmented’, with different aspects of bone that have been historically studied by different disciplines, such as medicine, biology, chemistry, physics, engineering, and material sciences. Each of them applied a variety of experimental and computational techniques (see Fig. 2) to get an insight into the bone properties and to unveil the fundamental mechanisms involved in bone tissue and its age-driven degradation. Nevertheless, communication among different disciplines, each one speaking its own language, is not always easy. An effort towards a thorough multidisciplinary approach, combining the results obtained from vastly different experimental and theoretical studies, is broadly instrumental to build a clear picture of bone aging and to get a profound understanding of aging-related alterations and diseases. For this purpose, in this paper we review the most prominent results regarding all aspects of bone aging, with a multiscale and multidisciplinary approach, to give the reader a wide view of the topic. (The present work mainly focuses on human bone. Through the dissertation, results coming from other animal models are reported when necessary to clarify some aspects of bone aging. Most of the reported studies on osteoporosis involving animals, especially species characterized by short life spans (such as mice) concern ovariectomized specimens or subjects fed with low calcium intake to mimic osteoporotic conditions in humans^11,12^. Nevertheless, the reader should keep in mind that bone could be considered a universal natural material, with similar composition among vertebrates and where significant differences across species arise mainly as architectural variations at the upper micro- and macro-scale, as an adaptation to specific body structures and movement requirements of each animal^13^. For this reason, several results on bone aging mechanisms obtained in animals maintain a general validity). Each section deals with a precise length scale (nano, micro, and macroscale, respectively), and each subsection delves into prominent changes observed in bone structure with age at that scale. Together with the results, each subsection contains details on the most common techniques employed to obtain them. We combine, in each subsection, results coming from different techniques, giving an idea of how each problem could be tackled in a multidisciplinary way. Open questions and unresolved issues are pointed-out, with some perspective on possible future research.

## Nanoscale structure of bone

At a nanoscale level, bones are made of several components that chemically and physically interact, imparting bones the mechanical and structural properties they need to sustain bodies, protect organs, and to respond to internal and external stimuli. At this length scale, the inorganic part is composed of hydroxyapatite (HAP) and water. The organic part is formed by collagen molecules and non-collagenous proteins (NCPs), such as osteopontin, osteocalcin, and proteoglycans.

Hydroxyapatite crystals and collagen molecules organize themselves into a precise structure, the fibril (see Fig. 1). The fibril is composed of collagen molecules aligned along their long direction, with some gaps, which harbor most of the HAP platelets. A precise periodicity exists in mineralized fibrils, called the D-period, on the scale of ≈67 nm. The D-period is composed of two parts, the overlap and gap regions; the gap is the space where hydroxyapatite fills most of the interfibrillar voids^14^. For many years, a linear packing model, with a simple lateral repeat of the fibril array has been considered for fibrils. More recent studies assume that the 67 nm periodicity, which determines the two-dimensional stacking, directs the three-dimensional pattern, generating a twisting crystalline structure in fibrils^10^. The mineralized collagen fibril is typically 50–200 nm in diameter^15^. Even if collagen and hydroxyapatite are the prominent bone building blocks at the nanoscale, the role of NCPs should not be underestimated. This group of proteins represents ~10% of bone extracellular matrix organic components^16,17^ and is located at the collagen-apatite interface^18,19^.

### Changes in hydroxyapatite platelet size during aging

Bone growth, especially for what concerns the mineral part, is a process that has been extensively studied: bone mineralization studies have been approached from both the experimental and computational point of views (for some recent examples see refs. ^14,20^). Bone mineralization starts from amorphous calcium phosphate deposited between collagen fibril molecules. From the mineralization nuclei, platelets of poorly crystalline carbonated apatite form both within the collagen fibrils (intra-fibrillar) and along their length (extra-fibrillar)^6^. In particular, extra-fibrillar HAP is thought to represent 75% of the mineral present in bone^21^. The HAP crystals, confined inside and in-between the collagen fibrils, grows and aggregates forming tiny crystals with a platelet shape. Their dimensions are in the range of tens to hundreds of nanometers, with the long axis usually aligned with the collagen fibrils^22–25^. The platelet shape of HAP, in particular its thickness (ranging from 1 to 7 nm in humans), has the advantage of maximizing the surface area, giving bone mineral a high biochemical and physiological reactivity^23^. This is of primary relevance considering that bone mineral is the main source of calcium for the entire organism, and this life-essential chemical element should be stored in an efficient way to be biologically available. Besides the biochemical role, HAP crystal shape and size also play a crucial role in the mechanical properties of bone nano-composites, as shown in previous computational studies which analyzed the effects of HAP nano-confinement^26,27^. It has been observed, for example, that the size of HAP crystals can influence the failure behavior at the nanoscale level, especially with respect to the stress distribution and crack propagation^28^. This is true not only for HAP crystals alone, but also for the collagen-HAP composite, where the nanoconfined size of HAP platelets enhances their strength, stiffness, and their capacity to dissipate mechanical energy^26^, and can influence the mechanical properties of interface between collagen and HAP^27^. Therefore, understanding the impact of aging on the size of HAP platelets in bones becomes a crucial issue to deepen our understanding of bone mechanics at the nanoscale level. Whether and how the crystal platelets change with age is an open and still debated issue.

**Fig. 3:**
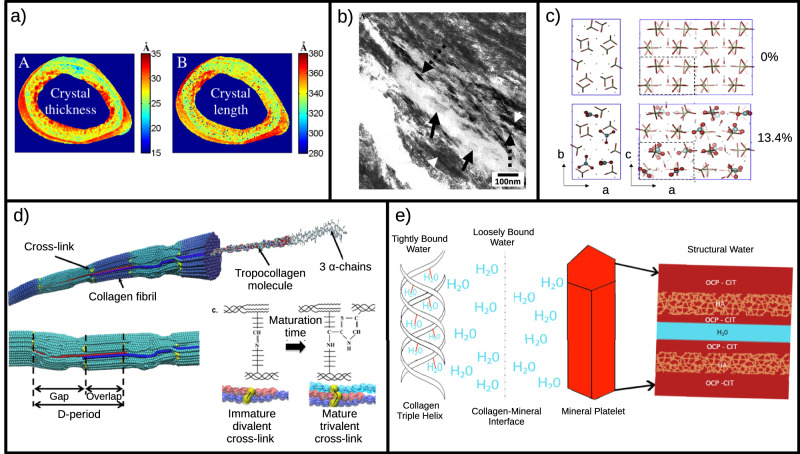
Different techniques to study bone aging at the nanoscale. **a** Map of crystal dimensions (respectively thickness and length) inside a healthy mature rat femoral cortex obtained with small- and wide-angle X-ray scattering (SAXS and WAXS) experiments from ref. ^25^; **b** TEM micrographs of longitudinally-sectioned mineralized collagen fibrils in human normal trabecular bone. Distinct individual apatite crystals are seen with plate-like shape (arrows) and tablet-like shape (representing plates on edge) (dotted arrows). Reprinted from Bone 33 (2003) 270–282, Matthew A. Rubin et al., “TEM analysis of the nanostructure of normal and osteoporotic human trabecular bone”, Copyright 2003, with permission from Elsevier^24^; **c** Schematic showing B-type carbonate substitution with atoms explicitly shown, for phosphate groups (only bonds shown) in the MD computational model, for different amounts (shown as wt%) of carbonate substitutions. Biomaterials 127 (2017) 75–88, Alix C. Deymier et al., “Protein-free formation of bone-like apatite: New insights into the key role of carbonation”, Copyright 2017, with permission from Elsevier^45^; **d** Schematic of a computer-simulated collagen fibril. The characteristic gap and overlap region are clearly visible. Focus on the immature divalent and mature trivalent cross-links (top) and their representation in the molecular model (bottom). Reprinted from Bone 110 (2018) 107–114, Baptiste Depalle et al., “The different distribution of enzymatic collagen cross-links found in adult and children bone result in different mechanical behavior of collagen”, Copyright 2018, with permission from Elsevier^47^; **e** Schematic representing the different locations of water inside HAP-collagen nanocomposites in bone. Water can be tightly bound to collagen triple helix, loosely bound at the interface between collagen and mineral, and present between the mineral platelets. Reprinted from Bone 120 (2019) 85–93, David B. Burr, “Changes in bone matrix properties with aging”, Copyright 2019, with permission from Elsevier^6^.

From an experimental point of view, measurements of HAP platelet dimensions have been performed on mouse^25,29^, bovine^23^ and human bone samples^22,24,30^ using a variety of techniques. The most widespread techniques are X-ray diffraction studies (for an example of data that can be obtained with this method, see Fig. 3a), Fourier-transform infrared spectroscopy (FTIR)^25,31^, and electron microscopy^24,32^. Yet, no consensus about how HAP platelets change in dimensions during aging has been reached, because of limitations of indirect measurement techniques (i.e., HAP crystal size changes could be a hypothesis to explain a variation in mineralization, but not the only factor that plays a role) and because of the very limited number of bone samples usually considered in each study. To give an idea to the reader of the state of the art and of the flourishing debate in this experimental field, we will summarize some significant findings. The experimental work of ref. ^29^ analyzed femurs from young adult (3-month old), middle-aged (8-month old) and aged (24-month old) Sprague–Dawley rats through Raman microspectroscopy. From the analysis of Raman spectra they observed a slight increase in crystallinity in old rats compared to young ones, suggesting a more ordered crystal lattice in the aged group. In addition, they measured a higher value of mineralization (relative amounts of mineral and organic matrix) in the aged samples with respect to the young ones. Those two aspects, increased mineral-to-matrix ratio and increased crystallinity could be explained by increased sizes of HAP platelets in aged rats^29^, a conclusion also reported in the recent review on changes in bone matrix properties with aging by ref. ^6^. Other authors, instead, found no significant differences in HAP platelets dimensions between young and old individuals^24,33^. This observation was reported from the Transmission Electron Microscopy (TEM) bright field analysis performed by Rubin et al. on six samples of human trabecular bones, three coming from young individuals and three from osteoporotic, aged ones^24^ (see Fig. 3b). Other authors, instead, observed a slight decrease of the HAP unit-cell volume associated with age: this is the case of a recent study by Foley et al. based on X-ray diffraction of bone samples coming from human skeletons buried in an old cemetery^30^. With this technique, the authors measured both the lattice parameters and the crystallite size, obtained as coherent diffracting domain inferred from the peaks of the X-ray spectra. Nevertheless, the measures of the crystallite sizes did not show a clear trend: in some bones, for example in the sternum, HAP platelets are larger in the aged sample; in other cases (such as the clavicle), they are shorter in the *c*-axis direction with respect to the young ones; yet in other bones the size is the same for both the young and aged HAP platelets. The authors pointed out a difficulty that is common to all the measurements conducted on HAP platelets through X-ray techniques: peaks broadening in X-ray spectra, due to the poorly crystalline nature of bioapatite, complicates the interpretation of crystallite size, computed as coherently scattering domain size obtained using the Scherrer equation^34^. Nevertheless, more detailed information has been obtained from X-ray diffraction about the dimension of the unit cell of bioapatite mineral. Foley et al.^30^ observed that the unit cell volume of bioapatite shrinks with age, due to a reduction of the *a* and *b* lattice parameters (see Fig. 3c), whereas the *c* length remains relatively constant with age. This confirms previous results obtained by ref. ^22^ via X-ray diffraction studies on a wider bone sample, composed of human iliac crest of 87 individuals aged 0–90 years, that revealed how lattice volume of bone apatite decreases with age. Moreover, it is consistent with the XRD measurements of ref. ^35^ that found lower lattice parameters in human adult bone samples compared to fetal ones.

This reduction of lattice parameters is caused by chemical changes that occurs in bioapatite during aging, in particular by the increase in carbonate content due to substitutions in the crystal lattice (for further details see “Increase of carbonate substitutions in HAP crystal lattice during aging”). Whether and how the variation of lattice parameters, that has been reported thus far, has a direct effect on the dimensions of the HAP nano-platelets present in bone is still unclear. As mentioned above, consensus on how the aging process affects the dimensions of HAP crystals inside bones has not been reached yet and an experimental study on a statistically significant number of young and old samples is still missing. X-ray studies have been the most popular technique to investigate crystal properties, thanks to the ease of the sample preparation process, but they have also shown some limitations in the spectra interpretation due to the low crystallinity of biological apatite. On the other hand, measurements of single HAP platelets from old and young bones could be performed with transmission electron microscopy (TEM) to highlight the modifications that aging could cause at a single platelet level. In that case, however, the bottle-neck process is sample preparation, since it requires particular attention in separating the mineral from the organic part of bone, while avoiding damage to the nano-crystals.

### Increase of carbonate substitutions in HAP crystal lattice during aging

The studies on the characteristics of hydroxyapatite platelets in bones also highlight a change in the chemical composition of the mineral during the aging process. The apatite structure is particularly favorable to chemical substitutions inside the crystal lattice. The most abundant substitution is carbonate (CO_3_)^−2^, which constitutes as much as 5–9 wt% of bone HAP^36^. In particular, there are two main types of substitutions: type A, that consist of substitutions of carbonate ions (CO_3_)^−2^ for hydroxyl ions (OH)^−^, and type B, that consist of substitutions of carbonate ions (CO_3_)^−2^ for phosphate ions (PO_4_)^−3^. In both cases, the charge is not balanced, and vacancies are introduced in the hydroxyl and calcium sites, to provide local charge compensation and increase the stability^37^. Further details about the chemistry and physics of the substitutions, their energetics, and how they modify the chemical bonds inside the crystal lattice can be found in previous theoretical studies based on quantum mechanics models^38,39^ and chemical studies on synthetic carbonated apatites^40,41^.

Several experimental studies have shown that the number of such defects in the mineral lattice increases with age and in the case of bone diseases such as osteoporosis^29,33,40,42^. Akkus et al.^29^, comparing femurs from Sprague-Dawley rats of different age groups, reported an incidence of carbonate substitutions in the aged group that was 17% greater than that measured in the young group. The study of ref. ^33^, instead, focused on the comparison between osteoporotic and non-osteoporotic bones, choosing samples coming from osteoporotic patients who had suffered fragility fractures at the femoral neck, and consequently required hip replacement surgery (thus considering particularly compromised bone tissue), and from healthy donors as control group. Based on XRD and FTIR analysis, the authors observed an 11% increase of the carbonate to phosphate ratio for the fractured osteoporotic material compared to the non-fractured one^33^. In addition, the review by ref. ^42^ described the reported trend of increasing carbonate to phosphate ratio with age in several animal models such as C57BL/6 mice^43^ and baboons^44^. Nevertheless, also on this crucial point, a broad consensus has not been reached about the precise impact of aging on the chemical composition of bone mineral. The recent FTIR measurements by ref. ^30^ suggest, instead, a decrease of carbonate content with age. Systematic compositional analysis to quantify the carbon content of each sample should clarify the issue and help resolve whether carbonate content varies with age. However, from the experimental side, the main obstacle to this straightforward approach is the difficulty of distinguishing between carbon compounds residing in the organic component of bone and carbonate in the apatite crystal structure^30^.

As discussed above, the presence of carbonate ions (CO_3_)^−2^ in the structure of hydroxyapatite modifies its lattice parameters and plays an essential role in regulating bone mineral morphology, decreasing the atomic order, coherent domain size, and surface energy^45^. These chemical changes have a significant impact on the mechanical properties and response of the bone mineral. Furthermore, the change in mechanical properties at the single crystal level should be considered as a possible cofactor contributing to the overall decrease in bone toughness observed at higher length scales with aging and osteoporosis. A recent study on synthetic apatites with different carbonate levels has experimentally determined apatite elastic properties, using in situ hydrostatic pressure loading and synchrotron X-ray diffraction. The measurements revealed that the increase of carbonate levels reduces the bulk modulus and the elastic strain ratio, likely as a result of decreased bond strength due to (CO_3_)^−2^ substitutions^46^. These recent experimental results are of particular interest from the perspective of studying the variations in mechanical properties of bone nano components during aging. For this reason, the role of carbonate substitutions occurring in bone hydroxyapatite during aging has been investigated also from a numerical point of view, through molecular dynamics (MD) simulations. Deymier et al.^45^ studied the effect of an increasing percentage of B-type carbonate substitutions (see Fig. 3c). They show an almost linear decrease in elastic moduli as a function of increasing carbonate concentration. Such studies are of primary importance to investigate, in a systematic and quantitative way, the effects that the increased number of carbonate substitutions have on the mechanical properties of bone. The controlled environment of simulations allows the variation of a single parameter at a time (in this case the carbonate substitutions percentage), disentangling effects that are difficult to be studied independently in experiments, and making it possible to perform direct mechanical testing at nanometer scale. It is important to note that, thus far, comparison with experimental results has been done with artificial apatites, that can be synthesized controlling the level of carbonate substitutions. From an experimental point of view, it would be of extreme interest to obtain results similar to Wingender et al^46^, but on biological bone samples characterized by different levels of carbonate substitutions. Of course, to achieve this goal, some technical difficulties need be overcome, regarding sample preparations and the above mentioned problem of precise quantification of the carbonate levels.

### Enzymatic and non-enzymatic cross-linking in bone

At the nanoscale, collagen cross-links, bonds that form within and between collagen molecules, are critical for modulating bone’s material properties. We distinguish between two different types of collagen cross-links: enzymatic and non-enzymatic ones. Enzymatic collagen cross-links form intermolecular covalent bonds between specific amino acids of tropocollagen helices. To form a mature and stable enzymatic cross-link, two stages are required. Initially, as the collagen molecules are released from the cell, the lysyl oxidase enzymatically modifies the lysine on specific sites. These lysine products (allysine, hydroxylysine and hydroxyallysine) link two collagen molecules together (such as hydroxylysino-norleucine (HLNL) and dihydroxylysino-norleucine (DHLNL)), in their immature divalent form. Reacting with different amino acids, these immature cross-links can further mature into a trivalent (or tetra-valent) form to connect two to three collagen molecules (such as pyridinoline (PYD) and deoxypyridinoline (DPD))(see Fig. 3d)^47^. Non-enzymatic collagen cross-links, instead, initiate from the glycation process and are named advanced glycation end-products (AGEs). From the chemical point of view, many different AGEs exist, such as vesperlysine, pentosidine, carboxymethyl-lysine, glucosepane, imidazolone, and furosine; nevertheless experimental studies on the effect of AGEs in bone tissue are based on quantitative measurements of just two AGE markers: pentosidine (the most widespread) and carboxymethyl-lysine (CML), that are easy to be accurately measured^6^.

Some enzymatic cross-links have shown changes in their quantities during aging. For example, histidinohydroxylysinonorleucine (HHL) has a rapid increase at birth followed by a gradual increase with aging^48^, and a recent study shows that there is a decline of immature enzymatic crosslinks during aging^49^. However, overall, the collagen cross-links formed by enzymatic processes do not appreciably change in number and properties with aging, are very stable^6,50^ and when they reach the mature, trivalent form, contribute to improve bone fracture toughness^51^. On the other side, significant amounts of AGEs accumulate in proteins with long half-lives, such as collagen, during aging^52^. More specifically, cross-links formed by non-enzymatic processes, measured through pentosidine content, show a significant increase in bone matrix with age^50^. Despite the well-established accumulation of AGEs in proteins contained in tissue of individuals affected by age-related chronic diseases, it is not yet clear whether AGEs are a cause or a consequence of aging^52,53^.

Even the impact that AGEs have on collagen mechanical properties has not been fully understood. While enzymatic cross-links form in very specific locations at the ends of the collagen molecules, non-enzymatic ones form in the middle of collagen fibers^6^. This could reduce collagen fiber deformation plasticity, as observed in in vitro ribation/glycation models^54^, making the tissue more brittle and contributing to reduced bone strength and toughness^6^. This is in agreement with some experimental studies on the role of collagen cross-links on mineralized fibril mechanical properties, conducted with small- and wide-angle X-ray scattering (SAXS and WAXS)^55^. The study of ref. ^55^, carried out on a group of patients with osteoarthritis, revealed an enhancement in the collagen scattering with age, that could be explained by an increased level of cross-links, responsible for collagen fibril stiffening. Experimental mechanical tests performed on denatured bone samples, instead, indicate that the stiffness of the collagen network decreases with age, irrespective of the increased AGEs accumulation^50^ and that bone collagen connectivity does not correlate with pentosidine content^56^. The experiments performed by refs. ^50,56^ are based on pentosidine measurments to quantify AGEs, and this could be a significant limitation since it represents only a possible biomarker of non-enzymatic cross-links, it is unclear whether the amount of pentosidine is proportionally related to the amount of the other advanced glycation end products^50^, and pentosidine content measured in aged human bone are small compared to the concentration of all of the covalent, enzymatic crosslinks found in bone^57^. Regarding the impact of age on collagen mechanobiology, recent studies also reported how the accumulation of AGE cross-links could promote the force transfer through the collagen backbone, instead of through friction between collagens (sliding), forcing the collagen to lose its ability to dissipate energy, and resulting in abrupt failures^58^. The introduction of AGE cross-links also leads to randomly located stress concentrations, due to their spontaneous formation sites, and might interfere with some naturally designed fracture mechanisms, for instance, the sacrifice of the weak bonds^59^, which diminishes the toughness of collagen fibril.

The mechanical implications of changes in collagen cross-links during aging deserve further investigation, to clarify the role of non-enzymatic cross-links. Undoubtedly, nanoscale collagen cross-links (both enzymatic and non-enzymatic) can play a role in influencing the mechanical properties and biological processes of bone even at higher length scales. At the cellular level, AGEs have been observed to interfere with integrin-mediated attachment of osteoblasts (cells specialized in bone construction) to a type-I collagen matrix^60^. Again, zooming out from the nanoscale upwards, it is interesting to notice how the formation of mature enzymatic collagen cross-links effectively turns the individual tropocollagen molecules into a large interconnected polymer structure^47^, which changes its plasticity when AGEs accumulate with age. This has a direct effect on mechanical properties, since connectivity and plasticity influence the fracture behavior of the collagen network^61^.

Besides a direct impact on collagen mechanical properties, collagen cross-links modifications with aging could also affect collagen degradation, influencing the bone remodeling process. A recent work of ref. ^62^ investigates how the changes undergone by collagen due to aging influence its degradation by matrix metalloproteinases (MMPs), a process that plays a crucial role in osteoporosis. The authors reported how aged collagen fibers (characterized by an increase in AGEs and mineral deposits) show a significantly reduced degradation with respect to the native fibrillar collagen. These observations suggest how aging modifications of collagen could block the degradation by either masking appropriate cleavage sites or by preventing the protease-generated partial unfolding because of increased stiffness.

A promising approach that could help in elucidating the direct effects of collagen cross-links on the mechanical properties of bone at the nanoscale is the one employed by ref. ^47^. The authors have implemented a coarse grain numerical model of collagen fibrils, to study the impact of enzymatic cross-link maturation from childhood to adulthood. It would be of extreme interest to apply the same approach to non-enzymatic cross-links, to characterize the effect of AGEs accumulation on nanoscale mechanical properties in silico. An excellent starting point for building in silico simulations of collagen is the recent online tool ColBuilder^63^, that include full-atom models of cross-linked collagen fibrils for 20 different species, obtained integrating the low-resolution structure of collagen fibril available from X-ray fiber diffraction with high-resolution structures of short collagen-like peptides from X-ray crystallography and mass spectrometry data^64^.

### Redistribution of water in bone during aging

Despite the popular notion of bone as an extremely solid and compact material, water is a fundamental part of bone tissue and represents 10% of bone’s relative composition^30^. At the nanoscale, inside the composite structure of bone, water molecules are present at different locations with a variety of biological, chemical, physical, and mechanical functions. In particular, water occupies free spaces in bone tissue or is bounded to collagen molecules or HAP mineral. The aging process has an impact on bone water, not only reducing its overall content in aged bone (dropping to 5% of bone’s relative composition), but also subjecting it to a redistribution inside bone tissue. Specifically, with aging there is a significant relocation of water from HAP-collagen interface, a loosely bound compartment, to the more tightly bound compartment, in which water molecules strongly bind to collagen triple helices (see Fig. 3e)^6^.

This has an impact on the mechanical properties of bone nano-composites. Previous computational studies on collagen-HAP nanocomposites have shown how hydrogen bonds could act as sacrificial bonds, facilitating protein sliding on the mineral surface in a discontinuous way, thus increasing the energy required for failure. Hence, water at the HAP-collagen interface acts as a “lubricant layer”, promoting the H-bond formation and leading to a general increase in the mechanical properties^26^. Besides, it enhances the shearing of collagen over hydroxyapatite, decreasing the direct coupling between collagen and mineral phase and redistributing load among them^27^. The water tightly bound to collagen molecules, instead, acts to stabilize the tropocollagen triple helix, stiffening the collagen fibrils. The relocation of water from the HAP-collagen interface to the tightly bound compartment that occurs with aging could worsen the overall mechanical properties of bone nano composites.

Water also plays a role inside the hydroxyapatite mineral itself: hydroxyapatite and bioapatite are extremely hydrophilic and form complex amorphous hydrated layers on their surfaces^46^. Davies et al.^65^ proposed a layered structure with thin apatitic platelets sandwiched between octacalcium phosphate citrate (OCP-citrate) hydrated layers (again see Fig. 3e) and demonstrated how such a model structure could explain NMR and X-ray data of real bones, such as the plate-like shape of mineral crystals, the presence of significant quantities of bound water molecules and the relatively high concentration of hydrogen phosphate. It has to been noticed that the presence of water and citrate layers between the HAP platelets could regulate the mineral crystallinity, preventing the platelets from merging into single larger crystals^6^. For this reason, a deeper investigation of the effect of aging on the structural water present in HAP mineral could be of particular interest.

### Non-collagenous proteins in bone and their role in aging

Non-collagenous proteins (NCP) make up ~10% of the organic component of bone extracellular matrix, with collagen being the dominant component making up the remaining 90%^16,17^. Different types of bone may contain differing amounts of the same NCP; for instance cortical bone contains significantly more osteocalcin compared to trabecular bone, while osteonectin is present in higher amounts in trabecular bone^66^. Moreover, bone matrix-associated NCPs are not exclusively unique to bone matrix; for example osteopontin and osteonectin are expressed in a variety of tissue types^67^. Recent studies suggest that NCPs in bone matrix are multifunctional and likely behave synergistically to affect cellular processes, such as cell attachment, cell differentiation, and regulation of hydroxyapatite deposition^68^. But, NCPs are not only biologically active molecules that contribute to cellular processes; given their sizeable fraction in bone matrix, they contribute to structural and mechanical properties directly as well. NCPs are localized at the collagen-apatite interface^18,19^. As a result, they likely contribute to load transfer at several hierarchical levels in bone matrix^6,69^. The absence of both osteocalcin and osteopontin in genetically-modified mouse models lacking NCPs osteocalcin and osteopontin, for example, is associated with reduced fracture toughness of bone^70^. Overall, while the role of NCPs in bone is increasingly well-understood, we know relatively little about how the quantity of NCPs changes with age and how age-associated trends impact the matrix quality. Of the limited information that exists on NCP changes with aging, most is concerned with the two predominant NCPs in the bone matrix, namely osteocalcin (OCN) and osteopontin (OPN).

Osteocalcin is the most abundant NCP in bone, making up ~20% of all NCPs in bone matrix^71^. Secreted by mature osteoblasts and by osteocytes, it has high affinity to calcium, accelerating nucleation of hydroxyapatite and regulating early stages of bone healing. It also regulates the activity of osteoclasts and promotes bone resorption, as well as insulin secretion and glucose homeostasis^71–80^. There is evidence to suggest that as people age, the level of circulating osteocalcin in their bodies decreases^81,82^, leading to a decrease in and altered distribution of OCN found in the bone matrix. This phenomenon has been observed in both men and women^83^, as well as in experimental animals such as mice and monkeys^81,82^. The amount of OCN in bone varies depending on the mean tissue age of the bone, with osteonal bone containing up to 20 times more OCN than interstitial bone. In mice, a lack of osteocalcin has been linked to increased net bone formation, which results in higher trabecular bone volume and periosteal apposition^76^. A recent study has also identified a direct link between glycation of OCN with the formation of the AGE pentosidine, and the loss of bone toughness^84^.

Osteopontin inhibits mineral formation and delays nucleation; osteopontin-deficient mice present larger crystal size and an increased mineral content^85^. Osteopontin also regulates bone resorption through osteoclast attachment and migration^86,87^. Simultaneously it may behave as a mechanical bone matrix “glue” that contributes to intrafibrillar sacrificial bonds, allowing greater stress relaxation and deformation prior to failure^88,89^. There is some evidence to suggest that while the quantity of some NCPs, like osteopontin, may not change with age, their composition may change, potentially affecting matrix properties. Notably, phosphorylation of bone matrix proteins begins to decline in middle age (at age 55-75) and appears to decline by roughly 20% by age 80^90^. This effect is more pronounced in women^90^. Higher levels of phosphorylation are associated with higher bone toughness, while decreased phosphorylation with aging alters the negatively charged OPN molecule and extinguishes an energy dissipation mechanism characterized by OPN unfolding and replacement by calcium ions on the hydroxyapatite surface. In the absence of this energy dissipation mechanism, bone toughness is diminished, allowing cracks to propagate more easily^90^.

While some recent studies have considered effects of aging on NCPs in bone matrix, the mechanisms of aging associated with changes to the quantity and quality of NCPs in the bone matrix are still poorly understood (see Table 1 for an overview on known NCPs functions and variation with age). Future work can provide further insight into the effects of aging on less abundant NCPs; while their low content in bone matrix may diminish their direct impact on mechanical bone quality over time, indirect effects are still likely at play. Resolving the cause-and-effect matrix of functional triggers and consequences of NCP modifications with aging may offer new, yet unexplored pathways toward targeted therapies.

**Table 1:** Role of noncollagenous proteins and trends with aging

Non-collagenous protein (NCP)	Functions	Species	Trend with aging
Osteocalcin	favors HAP nucleation, regulates the activity of osteoclasts, promotes bone resorption, insulin secretion and glucose homeostasis	humans, mice, monkeys	*↓*^81,82^
Osteopontin	inhibits mineral formation, delays nucleation, regulates bone resorption through osteoclast attachment and migration	humans, mice	≈^90^^a^
Biglycan	function in bone unknown, may regulate mineral crystal size	mice	?^67,118^
Fibrillin-2	expressed by differentiating osteoblasts, role in bone formation through signaling	mice	?^118^
Osteonectin	high affinity for calcium and phosphate ions, may regulate growth and proliferation of mineral crystals	mice	?^67,118^
Periostin	influence on collagen crosslinking	mice	?^118^
Bone Sialoprotein	HAP nucleator, enhances mineralization	mice	?^67,118^
Matrix extracellular protein	function in bone unknown	mice	?^118^
Decorin	may regulate collagen fibril diameter and fibril orientation, has a high affinity to type I collagen	mice	?^67,118^
Aggrecan	prevents cartilage calcification, function in bone unknown	mice	?^67^
Versican	function in bone unknown, could allow for cell binding to HAP	mice	?^67^
Alkaline Phosphatase	role in HAP mineralization	mice	?^67^
Tetranectin	function in bone unknown, may be involved in matrix mineralization	mice	?^67^
Thrombospondin	function in bone unknown,could be important in collagen fibrillogenesis	mice	?^67^
Fibronectin	highly up-regulated by osteoblasts, role during bone development	mice	?^67^
Vitronectin	strong attachment to osteogenic cells, may prepare the matrix for mineralization	mice	?^67^
Dentin matrix proteins	HAP nucleator, when phosphorylated inhibits HAP growth	mice	?^67^

^a^Degradation by phosphorylation.

## Microscale structure of bone

At the microscale, we distinguish between the extracellular matrix of bone and the cellular component of bone. These two components are closely linked, since bone is a living material, subject to the continuous remodeling action of specialized cells. At this length scale, the single bone fibrils arrange to form bone fibers, in combination with extrafibrillar hydroxyapatite. Each fibril is surrounded by a crust of mineral 20−30 nm thick and packed together and aligned with other fibrils to form fibers of 3–7 μm in width. Fibers are composed of single bundles of fibrils or by the combination of many bundles^10^. The organization of bone tissue at the microscale already reflects functional requirements of cortical and trabecular bone. Cortical bone consists of mineralized collagen fibers organized into 3–7 μm thick lamellae, which are arranged into concentric structures, thus forming the osteon. The osteon consists of a cylindrical structure of 100–500 μm in diameter crossed, along its length, by the Haversian canal, through which blood vessels and nerve endings run. Trabecular bone consists of trabeculae of lamellar bone oriented and intertwined with each other to form cavities in which bone marrow is stored and preserved. The orientation of the trabeculae results from mechanical forces applied on the bone tissue.

Inside the bone tissue, there exists a cellular component that consists of osteoprogenitor cells, osteoblasts, osteocytes, osteoclasts, and bone lining cells. Osteoprogenitor cells, osteoblasts and osteocytes are consecutive stages of the same cell type, the pluripotent mesenchymal cell that differentiates in osteogenic direction, whereas osteoclasts derive from the monocyte-macrophage lineage. The bone lining cells are quiescent osteoblasts implicated in the coupling between the resorption and neoformation processes^91^.

Osteoblasts, primarily involved in the synthesis of the bone matrix and its mineralization process, are polarized cells with high synthetic activity, as shown by the well-developed endoplasmic reticulum, and Golgi apparatus, and numerous secretory vesicles containing pyrophosphatase and alkaline phosphatase^92,93^. Alkaline phosphatase, when activated, produces phosphate ions that combine with calcium ions present in the extracellular matrix. In contrast, the role of pyrophosphatase is not fully defined. Some studies have proposed that activation of this enzyme leads to the release of pyrophosphate from the diphosphonates, thus leading to their inactivation. In fact, diphosphonates with pyrophosphate groups can combine with hydroxyapatite crystals, inhibiting the mineralization process^94^. For the mineralization of the bone matrix, a contribution is also made by molecules directly produced by osteoblasts. Osteonectin and osteocalcin are two of the molecules that contribute to the mineralization process: osteonectin promotes the nucleation of hydroxyapatite crystals, while osteocalcin inhibits the precipitation of calcium phosphate^95^.

The production of bone matrix and its subsequent mineralization occur following a precise orientation process:

- in a first step, the osteoblasts lay down the bone matrix from the side facing the pre-existing bone matrix;
- in a second step, deposition occurs along the entire circumference of the osteoblasts, thus distancing themselves from surrounding cells;
- during the final stages of matrix deposition, the osteoblasts slow down their metabolic processes by undergoing the transformation into osteocytes, while osteoprogenitor cells give rise to more osteoblasts;
- at the end of the bone tissue formation process, the osteoblasts close to the bone surface terminate their activity, resulting in morphological changes such as reduction of organelles and flattening of the cell, thus transforming into the cell phenotype of bone lining cells^96^.

Osteoblasts play a critically important role in the bone remodeling processes, by triggering bone matrix resorption through both direct and indirect mechanisms. In fact, osteoblasts also produce and secrete proteolytic enzymes that possess the ability to break down organic matrix components. One of these enzymes is the collagenase, which acts by removing the nonmineralized osteoid that lines the surface of the bone, thus allowing the osteoclast cellular component to adhere to the mineralized matrix and to break it down^97^.

Osteoclasts are multinucleated cells formed by the fusion of mononuclear progenitor cells, which in turn derive from hematopoietic precursors of the granulocyte-macrophage lineage (CFU-GM). In osteoclasts, the cell domain in direct contact with the bone surface is distinguishable into two zones: the clear zone, site of adhesion to the bone, and the ridged zone, characterized by the presence of numerous dense cellular extrusions^98^.

Osteoblasts can indirectly regulate the processes of bone remodeling by acting on the differentiation of osteoclasts, the cells responsible for bone matrix resorption. Osteoblasts secrete the cytokines RANKL (Receptor Activator of Nuclear Factor kB Ligand) and osteoprotegerin (OPG), whose release is balanced to maintain proper resorption activity by osteoclasts^99^. The cytokine RANKL, belonging to the superfamily of TNF (Tumor Necrosis Factor), is a protein localized at the membrane level of osteoblasts and their precursors. RANKL binds its receptor, RANK, which is expressed on the cell membrane of pre-osteoclasts. RANKL-RANK binding triggers the processes of differentiation toward the osteoclastic phenotype^100^. The main antagonist for RANKL activity is OPG, also expressed by cells of osteoblastic lineage, which competes with RANKL for binding to the RANK receptor. The RANKL/OPG ratio plays a key role in the regulation of bone resorption processes, so that ratio fluctuations can lead to the onset of bone pathologies due to a non-physiological regulation of the bone resorption process^101^.

The osteocytes are cells orchestrating these remodeling processes through biomechanical mechanisms (see “Canalicular network and fluid pressure acting on osteocytes”). Several computational models proposed in recent years have demonstrated the fundamental role of mechanical loading as a powerful and stable regulator of the biochemical processes underlying the mechanisms for maintaining bone architecture^102^. The mechanisms of perception of mechanical load change include deformation changes at the level of bone tissue, hydrostatic pressure, and flow potentials generated by extracellular fluid. It was initially thought that extracellular flow potentials could be generated by electrokinetic effects associated with a micropore system connected to the collagen-apatite structure^103^. Subsequent studies showed that the porosity was attributable precisely to the lacunar channels and that therefore these channels are the site from which flow potentials start^104^. Flow potentials are the electrokinetic effects that can regulate the movement of ions such as calcium across cell membranes. One of the earliest theories of fluid flow proposed that osteocyte activation was due to the flow of extracellular fluid along the dendritic processes of osteocytes. In vitro experiments demonstrated that osteocytes produce and secrete molecules in response to mechanical stimulation, upregulating the target genes of the Wnt/b-catenin pathway, the anabolic regulator of bone mass, and downregulating sclerostin, an antagonist of the Wnt signaling pathway^105^.

### Changes at the cellular level during aging

**Fig. 4:**
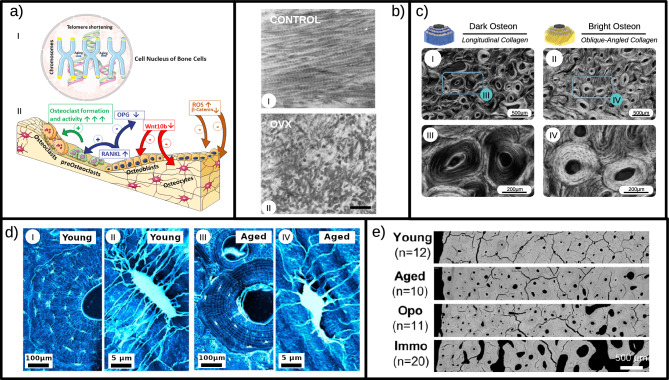
Different aspects of bone aging at the microscale. **a** Schematic representation of aging changes in bone cells. (**a**.I) In osteoblasts, osteoclasts, and osteocytes, telomeres decrease in length with each cell division due to the inability of the cell to fully replicate this region, and upon reaching a critical level of telomere length, cells undergo cellular senescence or apoptosis. (**a**.II) Altered rate of bone remodeling during aging. In osteoblasts, there is an increased secretion of RANKL, triggering the process of osteoclast differentiation (+), and a decrease of OPG, which competes with RANKL for binding to the RANK receptor inhibiting osteoclast formation and activity (−). The expression levels of all Wnt proteins, particularly Wnt10b, are significantly decreased in osteoblasts and osteocytes, thus reducing the bone anabolism (−). With aging, reactive oxygen species (ROS) increase and indirectly inhibit *β*-catenin, thus decreasing osteoblast formation (−). Subfigure (**a**) is built with Servier Medical Art; **b** Collagen fibrils coming from demineralized tibiae of a 10 months old female rat (control, I) and of an ovariectomized (used as an animal model for aging, II) specimen of same age viewed with electron microscopy. Fibrils in the ovariectomized case have an irregular arrangement in contrast to control. Scale bar is 0.5 μm. Reprinted from Kafantari et al. “Structural alterations in rat skin and bone collagen fibrils induced by ovariectomy.” Bone 26.4 (2000): 349–353, Copyright 2000, with permission from Elsevier^157^; **c** Circularly polarized light microscopy (CPL) experiments allow the visualization of osteons with different collagen fiber orientation: osteons composed of oblique orientated collagen fibers appear bright (II and zoomed in IV), while osteons composed of collagen fibers parallel with the osteon axis appear dark (I and zoomed in III). It has to been noticed that the two types of osteons coexist in bone samples of the same subject. Reprinted from ref. ^132^; **d** Changes in lacunocanalicular network and single osteocytes in young and aged individuals resulting from scanning electron micrograph of resin-embedded acid-etched osteonal bone performed by Milovanovic et al. The number of osteocytes lacunae in the young sample (I) is clearly higher than in the aged one (III), as well as the number of dendrites departing from a single osteocyte (II and IV). Reprinted with permission from Petar Milovanovic et al. “Osteocytic canalicular networks: morphological implications for altered mechanosensitivity.” ACS nano 7.9 (2013): 7542–7551. Copyright 2013 American Chemical Society^158^; **e** Comparison of cortical bone porosity between young, aged, osteoporotic (Opo), and immobilized (Immo) individuals in quantitative Backscattered Electron Images (qBEI) of human bone samples obtained using scanning electron microscopy (SEM) by ref. ^129^.

Osteoblasts, osteoclasts, and osteocytes have a limited lifespan, controlled by the number of replication cycles that decreases with age^106^, and which is presumably determined by the length of telomeres at the ends of their genes (see Fig. 4a.I). A telomere is a repetitive length of DNA and associated proteins that provide stability at the ends of chromosomes. However, telomeres decrease in length with each cell division due to the inability of the cell to fully replicate this region, and upon reaching a critical level of telomere length, cells undergo cellular senescence or apoptosis. In addition, levels of telomerase, the enzyme that can extend the life cycle, decrease with age^107^. External factors, such as UV rays and oxidative stress, also contribute to the aging process of bone cells by accelerating telomere shortening^108^. Numerous studies over the past decade have sought to elucidate the molecular regulation of telomere length. Of note, it was determined why humans have a relatively short telomere length of between 5 and 15 kb^109^, while in mice telomeres are about 50 kb long^110^, although humans have a much longer lifespan than mice. Evidence is emerging that a critical variable determining species lifespan is the rate of telomere shortening, rather than their initial length^110^. Specifically, human telomeres shorten at a rate of ~70 bp per year^111^, while mouse telomeres shorten at a rate of 7000 bp per year^110^, despite relatively abundant telomerase activity in mice^112^. This significant difference in telomeres shortening rate likely contributes to the difference in longevity between mice and humans^113^. Moreover, it has been shown that there is a non-linear shortening trend of telomere length across the human lifespan^114^ but similar studies have not been carried out in mouse models. In relation to bone tissue, telomere shortening, or telomerase deficiency, could be useful in monitoring the biological aging of bone cells. However, considering them as biomarkers to predict osteoporosis and fractures in the clinical setting is still preliminary. Indeed, the relationship between telomere shortening and uncoupling of bone remodeling processes observed in murine studies has not always been consistent with observational studies in humans^115^. These discrepancies may be due to some limitations of clinical studies, such as measuring telomere length in peripheral blood leukocytes rather than osteoblasts. Moreover, osteoporosis is a multifactorial disease, in which pathophysiological mechanisms other than age-dependent telomere shortening may prevail. For instance, as all cells show changes in gene expression and activity with age, bone cells may develop the inability to respond to mechano-transduction forces durig aging^116^. This increased susceptibility to mechanical damage leads to increased apoptosis and altered regulation of gene expression.

The aging process also affects bone mesenchymal stem cells (BMMSCs), reducing their proliferation and causing imbalanced differentiation, that significantly contribute to age-related bone loss^117^. Even changes occurring in bone extra-cellular matrix (ECM) at the nanoscale, could have direct consequences on bone cell activity and behavior. For example AGEs-modification of type-I collagen impairs the integrin-mediated adhesion of osteoblastic cells to the matrix^60^ and the presence of several NCPs could influence the adhesion between osteoblasts and osteclasts and ECM^67,118^.

### Changes in bone remodeling

The main cellular change impacting the skeleton is the altered rate of bone remodeling, the process by which osteoclasts remove existing bone and osteoblasts form new bone. In fact, as the number of osteoblast precursors decreases with age, the amount of bone deposited at each remodeling cycle decreases^119^. Cell aging in bone tissue is plausibly regulated by modifications of the signal pathway activated by proteins of the Wnt (Wingless-type MMTV integration site) family^120^. Comparing the expression levels of all Wnt proteins, their expression is significantly decreased in the bone tissue of aged mice compared with young mice^121^. In agreement with this, transgenic overexpression of Wnt 10b has shown to prevent bone loss in aged mice^122^. Notably, it has been suggested that oxidative stress also contributes to bone cell aging by interfering with the Wnt pathway, albeit indirectly. Indeed, reactive oxygen species (ROS) activate FoxO (forkhead box proteins), which in turn binds *β*-catenin, minimizing its effective concentration in cells and thus decreasing osteoblast formation^123^. In parallel, activation of PPAR-*γ* (peroxisome proliferator-activated receptor gamma) by ligands, generated by lipid oxidation, can negatively interfere with the Wnt pathway, contributing to the age-dependent decrease in osteoblast formation^124^ (see Fig. 4a.II).

Overall, these aging-induced cellular changes affect the rate of bone remodeling, the process by which osteoclasts remove existing bone (resorption) and osteoblasts replace it (formation). In fact, the amount of bone deposited at each remodeling cycle decreases due to a reduction in the number of osteoblast precursor cells, the number of mesenchymal cells from which they are derived, or a reduction in the lifespan of osteoblasts. There is also a parallel decrease in hematopoietic cells, which are precursors of osteoclasts. However, since the surface area available for resorption due to bone thinning also decreases, the net result is in favor of an increased rate of resorption in the bone remodeling process.

### Canalicular network and fluid pressure acting on osteocytes

Bone tissue is able to sense mechanical stimuli coming from the outside and to adapt itself in response, through the action of bone cells. Osteocytes are embedded in bone matrix, located inside specific micrometer-sized holes termed lacunae. Dendrites departing from osteocytes cross the bone matrix through narrow tunnels (roughly 300-nm-wide) named canaliculi, to connect with other osteocytes, osteblasts, and blood vessels^125^. This spatial organization gives rise to the lacunocanalicular network (LCN) that pervasively crosses bone tissue and within which an interstitial fluid is free to flow. The fluid moving in LCN inside bone tissue has been proposed to be responsible for passing mechanical signals to osteocytes. Indeed, deformations occurring through the bone matrix are too small to be directly sensed by bone cells and a further mechanism to communicate those mechanical changes is required. An external loading acts to deform the bone and the LCN network inside it, inducing a fluid movement through the canaliculi. This fluid flow could be sensed by osteocytes as a drag force sufficiently strong to trigger a mechanoresponse^126^. This hypothesis has been tested in the recent work of ref. ^127^, using controlled in vivo loading experiments on mouse tibiae. The authors used an interesting combination of a circuit theory model to calculate the fluid flow in realistic LCN network architectures, obtained from detailed microscopy images of bone sections, and in vivo microcomputed tomography (μCT) to directly measure formed and resorbed bone as a response to mechanical loading. This approach demonstrates a direct spatial correlation between predicted mechanoresponse, based on fluid flow patterns in the actual LCN architecture, and the measured mechanoresponse^127^.

Since LCN mechanotrasduction plays a key role in activating bone formation, it is of crucial importance to understand how the aging process affects this function. In a recent study using *μ*CT, the lacunae and canals in fibulae of female mice coming from two different age groups are compared: 5-month old (young adult mice) and 23-month old (old mice). These experiments give some clues of how LCN varies with age: older mice have lower canal volume density and show smaller and more rounded lacunae with respect to young mice^128^. Such morphological alterations in LCN with advancing age could potentially affect the ability of osteocytes to properly sense and respond to mechanical stimuli. The effects of aging on the lacunae, on the osteocytes themselves and on the canaliculi/dendrite connectivity are the object of a recent review^125^. A significant reduction (around 20%) in the number of lacunae per bone area is observed with advancing age, accompanied by a reduction in volume and a shift towards more spherical shapes of the lacunae themselves^125^. Observations on young and old mice also highlighted an increase in the percentage of empty lacunae (i.e., not occupied by an osteocyte) with aging^125^. Those lacunae left empty due to osteocyte death are subject to the micropetrosis phenomenon, being gradually filled with mineral. Furthermore, experiments on human and mouse bone also revealed a clear loss of osteocytes dendricity with increasing age, leading to a 30% decrease of the number of canaliculi^125^ (see Fig. 4d). These effects combined together lead to significant changes in canalicular fluid flow with aging, making the process of communication of mechanical loading less efficient and making the detection of microdamage less accurate. Furthermore, the alterations affecting the lacunocanalicular network could be associated with a specific pathology, as reported in the recent experimental study by ref. ^129^. Indeed, comparing bone samples of osteoporotic patients with samples coming from long-term immobilizated ones and from healthy ones as control, differences in LCN modifications have been reported^129^. For example, immobilization cases are characterized by lower osteocyte density accompanied by a higher proportion of empty lacunae, a symptom of increased osteocyte apoptosis with respect to the osteoporosis cases^129^ (see Fig. 4e). In this way, observing the LCN changes could also help one to better understand the mechanisms underlying bone degradation in different clinical conditions.

### Changes in collagen network

The aging process affects the collagen behavior not only at the molecular scale but at higher dimensions, impacting the complex hierarchical structure that collagen assumes in bone matrix. Collagen fibrils are assemblies of collagen molecules connected by covalent cross-links^130^. In this sense, the collagen structure can be considered to be a networked rope where each element of the array transmits force to the rest of the array via the lysine-hydroxylysine mediated crosslinks^131^. Furthermore, collagen fibrils themselves assemble into collagen fibers and then into lamellae and osteons, giving rise to a complex collagen network. Experimental studies at the beginning of 2000s identified a correlation between age-related changes in bone mechanical properties and collagen network integrity (see Fig. 4b). For example, the study by ref. ^50^ on thirty human femur samples coming from donors ranging from 19 to 89 years of age, measured both the mechanical properties of the bone samples, obtained via three-point bending tests, and of the demineralized bone extracted from the same specimens using an acid solution to remove the mineral phase and focus on collagen network characteristics. This approach revealed a significant decrease of failure strength, elastic modulus, and work to fracture of collagen network with increasing age. Furthermore, a significant correlation between mechanical integrity of the collagen network and work to fracture bone have been highlighted. More recently ref. ^56^ have performed similar mechanical tests on a wider and heterogeneous donor group composed of fifty-four individuals ranging from 21 to 101 years of age, performing also hydrothermal isometric tension (HIT) testing on decalcified bone samples. The collagen is heated, until it begins to denature; the melting process, in which its triple helix structure dissociated into a random amorphous coil conformation, drives the specimen to shrink. In HIT testing the collagen is held under isometric constraint, and the contractile force generated from the melting process can be measured. With this technique, the authors measure the collagen’s thermal stability, via the denaturation temperature, and network connectivity, measured as maximum rate of isometric tension generation. These experiments show a clear correlation between transverse fracture resistance of cortical bone and bone collagen network connectivity^56^. The limitation of these experimental studies stems from the delicate chemical procedures needed to separate the mineral part of the bone from the collagen phase. Decalcification, for example, can impact the hydration status of bone collagen, making the measurements less accurate.

An interesting approach to more deeply understand the implications of collagen connectivity on its mechanical properties comes from the recent study of Burla et al.^61^ that combines rheological experiments with computer simulations of collagen networks. The work focuses on disordered collagen networks, obtained by polymerizing collagen extracted from different species and various tissue sources at different temperatures, exhibiting a variety of collagen architectures. To consider a more widespread scenario in terms of connectivity, the authors add telopeptide sequences at the end of the collagen triple helix, to mediate intrafibrillar cross-linking. The authors demonstrate that the connectivity of collagen network, defined as the mean number of fibers meeting at a junction, is the main determinant of collagen fracture^61^. This result holds for isotropic collagen networks that could be found in skin and cartilage, while bone collagen is anisotropic with an ordered and hierarchical structure. Nevertheless, the way in which collagen fibrils and fibers are connected and interact with each other may have significant implications on bone mechanical properties.

### The role of osteons and different osteon morphotypes

Osteons are tubular structures, ≈100 μm in diameter, formed by aligned mineralized collagen lamellae that organize themselves into concentric layers. Osteons play a crucial role in deflecting and twisting cracks at the microscale, providing bone with extrinsic toughening mechanisms^9^, which contribute to toughness enhancement. When observed using circularly polarized light microscopy (CPL), two kinds of osteons can be detected, according to the collagen fiber orientation: “bright” ones, composed of oblique (±45^∘^) oriented collagen fibers and “dark” ones, composed of collagen fibers parallel with the osteon axis (see Fig. 4c). Recent studies revealed that these two classes of osteons not only differ in collagen fiber orientation, but also show differences in terms of composition and mechanical properties. “Dark” osteons have smaller and rounder lacunae, higher mineral-to-matrix ratio and carbonate-to-phosphate ratio, and higher elastic modulus with respect to “bright” ones^132^. Furthermore, it has been observed, through finite-element models (FEM) simulations and mechanical tests on 3D printed osteon-inspired samples, that mechanically “dark” osteons perform better under tension, while “bright” oseons perform better under compression^133^. Very interestingly, some of the characteristics of the osteons with parallel fibers ("dark”) are similar to the ones typically observed in aged bone. Yet they have been observed inside the bone of the same middle-aged individual, revealing a coexistence of these two kinds of osteons that play different mechanical roles inside the tissue: “dark” ones lead to higher stiffness and hardness while “bright” ones provide ductility and energy dissipation^132^. It would be of interest to study whether and how the number and spatial distribution of “dark” and “bright” osteons change with age, for example applying polarized light microscopy to a set of bone samples similar to the ones considered by ref. ^129^.

## Macroscale structure of bone

At the mesoscale, lamellae organize themselves into two distinct structures with different morphological and mechanical properties: the cortical and the trabecular bone. The cortical bone is a compact structure, usually found in the outer part of bones, formed by lamellae that regularly organize to form osteons around Haversian canals. The cortical bone is then composed of several osteons with interstitial lamellae around them (probably reminiscent of previous osteons), to form a compact and dense structure with high strength and stiffness. Trabecular bone, despite having the same chemical composition as cortical bone, has a completely different structure. It shows a spongy and porous structure, formed by small beams called trabeculae (composed of different layers of lamellae) that form a complex network rich in cavities. Trabecular bone is generally found in the inner part of bones and, due to the multiple orientations that the trabeculae can assume, it is particularly efficient in redistributing stresses. In a single bone there is a combination of these two tissues, with the cortical bone forming 80% of bone mass and the trabecular bone the remaining 20%. Nevertheless, due to its composition, the trabecular bone has a higher surface to volume ratio and is much more active from the metabolic point of view, being subject to more frequent remodeling. Mechanically, there is a combination of two different materials, the cortical bone with high strength and stiffness, able to resist high compressive and tensile loads, and the trabecular bone, more flexible and tough, able to absorb large amounts of energy. Considering a transversal section of a long bone, such as a human femur, proceeding from the external towards the internal part, one finds:

- The *periosteum*, a dense and thin membranous layer, mostly made of elastic fibrous material also containing blood vessels and nerves. In the inner part, it accommodates bone cell progenitors and osteoblasts.
- The *intracortical area*, the central part of cortical bone, with osteons and interstitial lamellae around them. The intracortical area is crossed by the lacunocanalicular network, with osteocytes inside it, and by Harvesian canals that allow the passage of blood vessels and nerves.
- The *endosteum*, a thin membrane that surrounds the medullary cavity. It consists of a layer of flattened osteoprogenitor cells and collagenous fibers.
- The *trabecular part*, which occupies the inner part of long bones. Between the holes of the spongy tissue, *bone marrow* is accomodated. Bone marrow is a semi-solid tissue formed by hematopoietic cells, marrow adipose tissue, and supportive stromal cells.

At this length scale, bone tissue could be observed in a direct way with imaging techniques such as Computed Tomography (CT) and Dual X-ray absorptiometry (DXA), and experimentally characterized through mechanical testing (e.g., tensile, compressive, three point bending tests).

### Changes in bone mineral density (BMD)

**Fig. 5:**
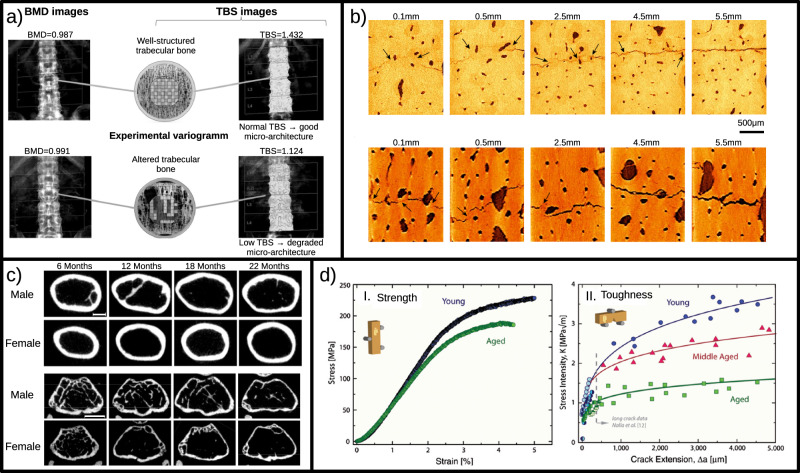
Studying effects of aging on bone tissue at the macroscale. **a** Dual X-ray absorptiometry (DXA) images of human spine allowing the comparison between Bone Mineral Density (BMD) and Trabecular Bone Score (TBS) scoring techniques. Two different patients with similar BMD could show different TBSs. Thanks to the experimental variograms, TBS takes into account the degradation of bone microarchitecture. Figure reprinted from ref. ^138^; **b** Two-dimensional computed X-ray tomographic reconstructions showing crack propagation in bone longitudinal direction. Each image represents a transverse section taken at different distances from the nominal crack tip (see numbers on top of each figure). The comparison is between young (top row) and old (bottom row) human bone samples. Differences in micro-crack dimensions and uncracked-ligament bridges (indicated by black arrows) are clearly visible. Reprinted from R.K. Nalla et al. “Effect of aging on the toughness of human cortical bone:evaluation by R-curves” Bone 35 (2004) 1240–1246, Copyright 2004, with permission from Elsevier^152^; **c** 2D cross-sectional images of mouse femurs obtained with μCT. Top rows show the femoral cortical bone in both male and female mice of different ages (6, 12, 18, and 22 months respectively). Bottom rows, instead, show femoral trabecular bone in male and female mice of different ages (6, 12, 18, and 22 months respectively). Scale bars are 1 mm. Figure adapted from ref. ^141^; **d** Mechanical properties of human cortical bone as a function of age obtained with three-point bending mechanical tests. (I) Stress–strain curves and (II) fracture–toughness R-curves show a decline of both strength and crack toughness with advancing age. Figure reproduced from ref. ^147^.

The most evident change that could be macroscopically detected in bones with aging, is the loss of bone mass. The estimation of bone “quantity” is of daily use in clinical practice, where it is usually measured as bone mineral density (BMD). The measure of the BMD is performed via Dual-X Ray Photon Absorptiometry (DXA), a technique developed in the mid-1980s, that uses highly collimated low-energy X-ray beams passing through soft tissues and bony parts of the body, captured by a detector. In this way it is possible to obtain an image from the exiting beam, where the intensity of the image is inversely related to the density of the traversed body parts^134^. Thanks to the low dose of radiation needed and the non-invasiveness of the procedure, BMD became very popular as predictor of bone resistance to load and fracture and is today the gold diagnostic standard for osteoporosis diagnosis. Population studies showed how in humans BMD increases during childhood and reaches a maximum value around 30 years of age both in men and women. After the peak BMD starts to gradually decline in men with advancing age. In women, instead, there is a more rapid decline of bone mass associated with menopause, that becomes more gradual with further aging. Despite its spread, BMD has some intrinsic limitations. First of all, it depends on the anatomical location of the sample^134^ and on the instrument calibration based on standard phantoms of known density that should be carefully chosen^135^. Moreover, BMD is an areal measurement, expressed in grams per square centimeter and because of its two-dimensional nature it cannot capture bone three-dimensional microarchitecture and distinguish between the cortical and trabecular tissue. For this reason, a new morphological parameter has been introduced to gain information on bone microarchitecture, the trabecular bone score (TBS). TBS is obtained from DXA, as BMD, and it is a textural measurement based on the experimental variograms of projected gray-level DXA images^136^. As seen in Fig. 5a, TBS is able to distinguish among patients with similar BMD, unveiling skeletal microstructure features often associated with aging or pathology^137,138^. The research of appropriate and highly predictive tools to assess bone quantity and quality at the macroscale level and to estimate fracture risks in clinical practice is a wide and continuously evolving field. Together with BMD and TBS, officially accepted in clinical practice, researchers proposed other instruments, for example the FRAX, a computer-based algorithm developed by the World Health Organization Collaborating Centre for Metabolic Bone Diseases that calculates fracture probability from readily obtained clinical risk factors^139^ and the BSI (Bone Strain Index), obtained from a complementary tool, based on Finite Element (FE) models, that assesses the resistance of bone to simulated compressive loads to improve the DXA accuracy^140^. Nevertheless, it is important to understand that each of those measurements on macroscopic bone features is not able to accurately predict fracture risk and could not reveal all the subtle changes occurring with age and disease in such a complex tissue as bone. It will be necessary to make a combined use of them and even to make an effort to connect those macroscopic changes with more fundamental processes occurring at lower length scales.

### Changes in morphological parameters measured with μCT

Widely used in bone research is the microcomputed tomography technique (μCT), an X-ray-based imaging tool that allows the production of high-resolution three-dimensional (3D) images composed of two-dimensional “slices” of a target specimen. With that technique, it is possible to measure features of bone architecture in the μm-mm scale, obtaining more detailed and quantitative information on cortical and trabecular tissue and their morphological variation with aging. In Table 2 we show some of the most significant morphological parameters that have been measured for bone tissue at the meso and macroscale, together with their variation with age as observed in previous experimental works on both humans and animals. Indeed, numerous studies have shown that mouse models, particularly the C57BL/6J strain, share with humans such features as ovarian follicle decline, irregular cycle, and steroid hormone fluctuations that influence bone. Therefore, they have been widely used in research on mechanisms responsible for aging morphological changes in the skeleton due to their small size and short lifespan (average of about 2 years). In male and female mice, the volume of trabecular bone in the long bones begins to decrease in parallel with a reduction in cortical thickness (Ct.Th) after about 6 months of age, with a higher degree of reduction in females^141^ (see Fig. 5c). μCT analysis of femurs showed the ratio between trabecular volume and total volume (BV/TV) decline by 63.8% in male mice and 80.8% in females from 6 to 22 months of age. In correlation with BV/TV reductions, parallel decreases in trabecular number (Tb.N) of 12.1% and 40.3%, respectively, were observed. Likewise, trabecular BMD was reduced by 27.4% in males and 41% in females. As for trabecular bone of tibia, BV/TV was decreased by 60.4% in males and 85.6% in females, associated with Tb.N decline of 24.6% and 47.6%, respectively. Trabecular bone of the spine showed similar reductions in BV/TV, accompanied by 19.1% Tb.N reduction in males and 42.5% in females^141^. The decline in BV/TV is most attributable to a decrease in the number of trabeculae, which may be accompanied by an increase in trabecular thickness, particularly in long bones. This may be due to an increase in the average thickness of the remaining trabeculae as the thinner ones are removed. However, trabecular thickness has been shown to increase due to a compensatory mechanism by the remaining trabeculae, which support increased mechanical loads as the thinner ones are reabsorbed^142^. It is still unclear whether, in the face of endocortical resorption and decline in material properties with age, periosteal expansion continues with aging in the mid-diaphyseal regions to maintain flexural strength of whole bone. In fact, some studies observed that there was no substantial increase in the cross-sectional area of cortical bone despite the continued loss of trabecular bone volume^142^.

**Table 2:** Macroscale characteristics of bone tissue from imaging techniques

Quantity	Description	Species	Trend with aging
Tb.BMD (mgHA/cm^3^)	trabecular bone density	mice/humans	*↓*^42,141^
Ct.BMD (mgHA/cm^3^)	cortical bone density	humans	*↓*^42^
Ct.CSA (mm^2^)	cross-sectional area of the cortical tissue	mice	≈ ^142^
Ct.Th (mm)	thickness of the cortical tissue	mice	*↓*^141^
BV/TV (%)	ratio between trabecular volume and total volume	mice	*↓*^141^
Tb.Th (μm)	trabecular thickness (avg)	mice	*↑*^142^
Tb.Th (μm)	trabecular thickness (avg)	humans	*↓*^42^
Tb.N (1/mm)	trabecular number ($Tb.N=\frac{1}{Tb.Th+Tb.Sp}$)	mice/humans	*↓*^42,141^
Tb.Sp (μm)	separation among trabeculae (avg)	humans	*↑*^42^
ConnD (1/mm^3^)	trabecular connectivity density (# of connecting paths)	humans	*↓*^42^
Ct.Po (%)	percentage of cortical porosity (segmentation)	humans	*↑*^42^
HC (μm)	Harvesian canal diameter	humans	*↑*^143^
OWT (μm)	osteon wall thickness	humans	*↓*^143^

The variation of bone morphological parameters with age has also been studied in human bone samples, confirming many trends observed in animal models. Loss of trabecular bone mass in humans starts from the third decade of life and in women is accelerated with menopause; in elderly individuals cortical bone (Ct.BMD) loss and increased cortical bone porosity (Ct.Po) are also observed. The main modifications induced by age in the trabecular tissue are the decrease of the trabecular number (Tb.N), thickness (Tb.Th), and connectivity (measured as connectivity density ConnD). In those processes, sex-specific differences have been observed, showing how the loss of trabecular bone mass in women is mainly due to the reduction of the number of trabeculae, while men lose bone mass prominently because trabecular thinning occurs. Furthermore osteoporotic patients show lower bone volume fraction (BV/TV) with respect to old normal individuals, with osteoporotic women showing even lower values of BV/TV with respect to osteoporotic men^42^. It has to been noticed how results on Tb.Th trends with aging differ among mice and humans, showing a thickening of the remaining trabeculae in mice and a thinning of the trabeculae in humans. Further investigation is needed to clarify this issue, to reveal different mechanisms involved in the two species. Going up in scale, μCT images on human cadaveric bone samples allowed the measurement of the morphological properties of the Harvesian structure present in cortical bone. Those measurements revealed how the Haversian canal (HC) diameter increases with aging while the osteon wall thickness (OWT) decreases significantly, with a direct impact on the bone’s ability to deflect cracks^143^.

For the sake of brevity, we only highlight the trend that each morphological parameter follows during aging, without further details on the numerical values typically assumed. The interested reader can find in the recent review by van der Bergh et al.^144^ a collection of updated numerical values of bone structure parameters coming from HR-pQCT for female and male human subjects and, when available, their variation with aging.

### Changes to mechanical properties

The idea of characterizing bone tissue at the macroscale, using load-displacement curves obtained from mechanical tests, as usual in an engineering study of a material, goes back to the ’70s^145,146^. Already from the first studies, an overall worsening in terms of mechanical properties with aging is revealed^146^. This trend has then been confirmed in later experimental works^147,148^ (see Fig. 5d). In the following, we briefly review the main variations that aging causes to bone tissue properties. Cortical and trabecular bones are usually tested independently, since their different structures imbue them with different mechanical properties, hence different loading curves^8^. Results have been summarized in Table 3.

**Table 3:** Macroscale mechanical properties of bone tissue

Quantity	Description	Technique	Trend with aging
E (GPa)	Elastic modulus	nanoindentation	≈ ^143^
W_*f*_ (kJ/m^2^)	Work to fracture (absorbed energy)	3-point bending test	*↓*^50,150^
J_*grow*_ (MJ/m^3^)	crack growth toughness (elastoplastic)	3-point bending test	*↓*^143^
J_*ic*_ (KJ/m^2^)	crack initiation toughness (elastoplastic)	3-point bending test	*↓*^143^
K (MPa$\sqrt{m}$/mm)	crack growth toughness (plastic)	loading test with notch	*↓*^152^
K_0_ (MPa$\sqrt{m}$)	crack initiation toughness (plastic)	loading test with notch	*↓*^152^
*σ*_*u*_ (MPa)	tensile ultimate stress (strength)		*↓*^147,150^ or ≈^156^
*ϵ* (% or adimensional)	tensile ultimate strain		*↓*^8^

#### Reduction of energy absorption with aging

The quantity of energy that a material sample is able to absorb gives an idea of material toughness and tells us something about the material’s ability to respond to external mechanical loads and adapt to them, prior to failure. More in detail, it is possible to estimate the work to fracture (W_*f*_) of a sample, defined as the work per unit area to break into two pieces an unnotched specimen loaded in bending or tension. From an experimental point of view, W_*f*_ is measured by computing the area under the load/displacement curve divided by twice the area of the fracture surface^149^. The first measurements of W_*f*_, performed on mm-sized cortical bone samples extracted from human donors of different age, already showed a decline of absorbed energy with advancing age. In particular, each decade of life seems to bring a decline of W_*f*_ of 8.7% of its value at 35 years^150^. Subsequent studies not only confirmed the decrease of work to fracture with increasing age, but also underlined differences between energy absorption in the preyield and postyield regimes. Indeed, the total elastic energy absorbed in preyield deformation (W_*fc*_) is almost constant for young and middle aged bone samples, showing a decline of almost 30% for elderly ones. The energy absorbed during postyield deformation (W_*fp*_), instead, decreases continuously with advancing age and correlates with the progressive deterioration of collagen networks’ mechanical properties^50^. Even in bovine samples tested under compression in the transverse and longitudinal directions, the subtended area of the stress-strain curves is significantly smaller for the adult specimen with respect to the young one, underlying the greater ability of young bone to absorb energy and to deform plastically without fracturing^151^. For trabecular bone, instead, the primary macroscopic mechanisms for energy absorption are fracture and buckling of trabeculae, allowing reduction of the stresses transmitted to the cortical tissue and the delay of the complete bone failure^145^. It is clear, therefore, how a decrease in the number of trabeculae (see paragraph “Changes in morphological parameters measured with µCT”) with aging has a significant impact on the decrease of the absorbed energy. Nevertheless, directly comparing the work to fracture data coming from different studies could be difficult because there is a dependence on the sample dimensions that should be taken into account. For this reason many recent works used the crack initiation and growth to asses bone sample toughness (see subsection “Decline of crack initiation- and crack growth-toughness with age”).

#### Decline of crack initiation- and crack growth-toughness with age

Mechanical tests performed on real bone samples show how fracture onset and propagation change with age both in cortical and trabecular bone tissue. Nalla et al.^152^ measured crack propagation in the longitudinal direction (parallel to osteons axis) of human cortical bones taken from donors from 34 to 99 years of age. The authors reported a significant decrease of crack initiation toughness with age (reducing in value by 40% from 40 to 100 years of age) and an evident decline of crack-growth toughness, that becomes almost negligible in the oldest samples. Furthermore, based on integrated use of synchrotron X-ray computed tomography (SRCT), a direct observation of the crack propagation and microstructure is possible (see Fig. 5b). This technique highlights how the number and size of crack bridges (uncracked portion of tissue separating crack fronts) significantly decrease with age, depriving bone of a crucial extrinsic toughening mechanism^152^. Similar results have been recently confirmed by nanoindentation tests performed by Yadav et al.^143^ on young and aged cadaver bones. They found that even in the transverse direction (perpendicular to osteons axis) both crack initiation and crack growth toughness are significantly lower in the aged group^143^. In this transverse direction osteons play a fundamental role in toughening the tissue deflecting the cracks^9^. Yadav et al.^143^ reported an increase in Harvesian canal diameters and a decrease in osteons wall thickness with age. These features weaken the osteon crack deflection ability, making the cracks initiate and grow more easily. In the trabecular tissue, instead, the crack initiation and propagation are less explored issues, due to high shape complexity. A recent work of ref. ^153^ applied cyclic compressive loading to human trabecular bone samples using contrast agents to directly observe the location and distribution of microscopic cracks and accumulation of submicroscopic cracks. Interestingly, the study revealed how damage distributes and how samples with thinner “rod-like” trabeculae, transversely oriented to applied loads, show enhanced fatigue resistance^153^. That approach of “coloring” damaged, microcracked regions was used to obtain insights into the mechanisms involved in fatigue resistance, but could be potentially expanded and exploited to study the variation of trabecular bone damage patterns with aging.

#### Reduction in tensile ultimate strain with age

In addition to the ability to absorb energy and resist crack initiation and propagation, bone tissue also loses plasticity and ability to deform with advancing age. From a mechanical point of view, this property could be assessed by measuring the tensile ultimate strain, defined as the strain value corresponding to the peak of the stress-strain curve. Tensile ultimate strain gives a measure of the maximum deformation that a material is able to sustain prior to failure. Morgan et al.^8^ reported a decline of tensile ultimate strain for human cortical bone of approximately 10% per decade, from a high of 5% strain at age 20–30 years to a low of less than 1% strain above age 80 years. This is an indicator of how bone becomes macroscopically less ductile and more brittle with aging, favouring the onset of fractures. Interestingly, in-situ SAXS measurements performed on human bone samples during tensile tests, have shown that for a given strain applied to the bone tissue, the strain carried by the collagen fibrils is significantly less (by some 25%), in aged bone compared to young^147^. This behavior is even more evident in diabetic bone in mice, where the ultimate strain of collagen fibrils is reduced by 40% respect to healthy mice^154^. Those facts suggest a key role of collagen fibrils in decreasing overall bone ultimate strain, becoming less deformable, probably due to the increasing presence of AGEs cross-links (see “Enzymatic and non-enzymatic cross linking in bone”)^147,154^.

## Remarks and perspectives

“There is plenty of room at the bottom” said the Nobel Prize physicist Richard Feynman showing a pin to his audience. With that famous quote he was explaining how behind a common object there is still a lot to uncover going down in scale, reaching atoms and subatomic particles and considering the fundamental interactions among them^155^. In a certain way, we aim to do the same with bone tissue research and treatment strategies for bone aging. This peculiar biological tissue has been the subject of many research works since decades, attracting the interest of various fields, ranging from medicine and biology to engineering and physics. Each discipline borrowed its techniques to unveil some aspects of bone structure, characteristic of the material and biological process involved in bone formation and remodeling.

As emerging from the previous sections, the aging process affects the bone tissue in many different ways, with repercussions at multiple length scales. Indeed, changes in the chemical structures (i.e., mineralization and chemical defects in hydroxyapatite) and molecular bonds (i.e., collagen cross-links) of bone components occur at the nanoscale, modifying the fundamental building blocks that constitute bone hierarchical structure. Besides, bone cell activity is significantly modified by aging. In particular, the number of osteoblasts precursors decreases, with a significant impact on bone remodeling process, especially on new bone formation, since the amount of bone deposited at each remodeling cycle decreases. Then, aging brings distinct changes to bone tissues, as observed at the micro- and macro- length scales with different imaging techniques, with a direct and measurable impact also on bone mechanical properties, which tend to decline with advancing age.

Even if each of those aspects has been investigated separately, through experimental or computational techniques, reaching in certain cases a good agreement among different studies and exhaustive explanations, there is still room for further scientific exploration. At the nanoscale level, how hydroxyapatite crystals change in dimensions and composition with age, how collagen cross-links vary and how non-collagenous properties behave with aging are open questions, still awaiting a definitive answer. Moreover, the investigation of links, connections and relationships among different aspects of bone aging occurring at different length scales could be particularly important. There is still a lot to unveil about how changes occurring at the molecular and cellular levels influence the macroscopic behavior of bone, in a direct or indirect way. Clarifying and quantifying how modifications of bone building blocks at the nanoscale translate to bone tissue properties is an extremely interesting area of research with many gaps in knowledge yet to be filled. This challenge requires a truly multidisciplinary approach, with the interpenetration of different skills and the joint use of different techniques. Even if challenging, that approach would be of high interest for a profound understanding of the fundamental mechanisms involved in bone aging, paving the way for the development of better diagnostics, and more efficient and targeted therapies for age-related bone diseases.
